# Lentivirus mediated silencing of Ubiquitin Specific Peptidase 39 inhibits cell proliferation of human hepatocellular carcinoma cells in vitro

**DOI:** 10.1186/s40659-015-0006-y

**Published:** 2015-03-19

**Authors:** Zeya Pan, Hao Pan, Jin Zhang, Yun Yang, Hui Liu, Yuan Yang, Gang Huang, Junsheng Ni, Jian Huang, Weiping Zhou

**Affiliations:** The Third Department of Hepatic Surgery, Eastern Hepatobiliary Surgery Hospital, Second Military Medical University, 200438 Shanghai, China; Department of Infectious Disease Control and Prevention, Shanghai Municipal Center for Disease Control and Prevention, 1380 West Zhongshan Road, 200336 Shanghai, China

**Keywords:** Ubiquitin Specific Peptidase 39, Lentivirus, Human hepatocellular carcinoma, Cell proliferation, Cell cycle

## Abstract

**Background:**

Ubiquitin Specific Peptidase 39 (USP39) is a 65 kDa SR-related protein involved in RNA splicing. Previous studies showed that USP39 is related with tumorigenesis of human breast cancer cells.

**Results:**

In the present study, we investigated the functions of USP39 in human hepatocellular carcinoma (HCC) cell line SMMC-7721. We knocked down the expression of USP39 through lentivirus mediated RNA interference. The results of qRT-PCR and western blotting assay showed that both the mRNA and protein levels were suppressed efficiently after USP39 specific shRNA was delivered into SMMC-7721 cells. Cell growth was significantly inhibited as determined by MTT assay. Crystal violet staining indicated that colony numbers and sizes were both reduced after knock-down of USP39. Furthermore, suppression of USP39 arrested cell cycle progression at G_2_/M phase in SMMC-7721cells. In addition, Annexin V showed that downregulation of USP39 significantly increased the population of apoptotic cells.

**Conclusions:**

All our results suggest that USP39 is important for HCC cell proliferation and is a potential target for molecular therapy of HCC.

**Electronic supplementary material:**

The online version of this article (doi:10.1186/s40659-015-0006-y) contains supplementary material, which is available to authorized users.

## Background

Human hepatocellular carcinoma (HCC) is the most popular type of liver cancer, which is mainly caused by viral infection [1-3]. It ranks as one of the top reasons of cancer related death all over the world [4,5]. The high rate of tumor recurrence and intrahepatic metastasis lead to poor prognosis of HCC patients [2]. To find out the molecules associated with HCC is very important for therapy of HCC [6].

Recently, RNA interference (RNAi) has emerged as a powerful tool to down-regulate the expression of target genes in mammalian cells and animals, especially in cancer cells [7]. Lentiviral vectors taking small hairpin RNA (shRNA) are found capable of silencing target gene expression with high specificity, stability and efficiency in a variety of human cancer cells including HCC cells [8,9,6].

RNA splicing plays key role in eukaryotic gene expression [10]. Mutations of splicing elements or changes of activities could affect tumor genesis and progression [11,12]. Ubiquitin Specific Peptidase 39 (USP39) is a 65 kDa SR-related protein in human cells involved in assembly of spliceosome, as well as a deubiquitinating enzyme without protease activity [13,14]. Although USP39 included the ubiquitin enzyme area but without de-ubiquitinating enzymes activity. USP39 plays an important role in the maintenance of mitotic spindle checkpoint function. The function is that it strictly controlled the activity of ubiquitin ligase. And only when all chromosomes are attached to the spindle in anaphase of mitosis, it will release the ubiquitin ligase activity [15]. As is known, the spliceosome consists of five small nuclear ribonucleoproteins (snRNPs), U1, U2, U4, U5 and U6, along with many non-snRNP proteins. Human USP39 protein is a tri-snRNP-specific protein, which is essential for recruitment of the tri-snRNP to the pre-spliceosome [16]. USP39 is highly conserved, with 65% homology to the yeast Sad1p. Sad1p is involved in the assembly of U4 snRNP to U6 snRNP and splicing both *in vivo* and *in vitro* [13,14].

The gene mutation of USP39 can cause that the mutation of retinoblastoma rb1 mRNA splicing is blocked, and leading to the occurrence of pituitary adenoma [17]. It showed that the down-regulation of USP39 gene can cause rb1 mRNA splicing abnormalities, which then leaded to downstream target genes e2f4 up-regulated in zebrafish. It is well known that e2f4 is a main regulator, it has the strong ability to cause tumor formation when it is overexpressed.

Previous studies found that down-regulation of USP39 could inhibit cell growth and colony formation of human breast cancer cells [18]. USP39 is also involved in the proliferation of prostate cancer cells and its SUMOylation is important for its function [19]. However, there is no report about the functions of USP39 in human hepatocellular carcinoma. In this study, taking advantage of lentivirus mediated RNAi, we inhibited the expression of USP39 in SMMC-7721 cells. We then analyzed the functions of USP39 in SMMC-7721 cell growth and colony formation. Furthermore, we checked the cell cycle progression after knock-down of USP39.

## Results

### Expression of USP39 was suppressed efficiently in SMMC-7721 cells by lentivirus mediated RNAi

**Figure 1:**
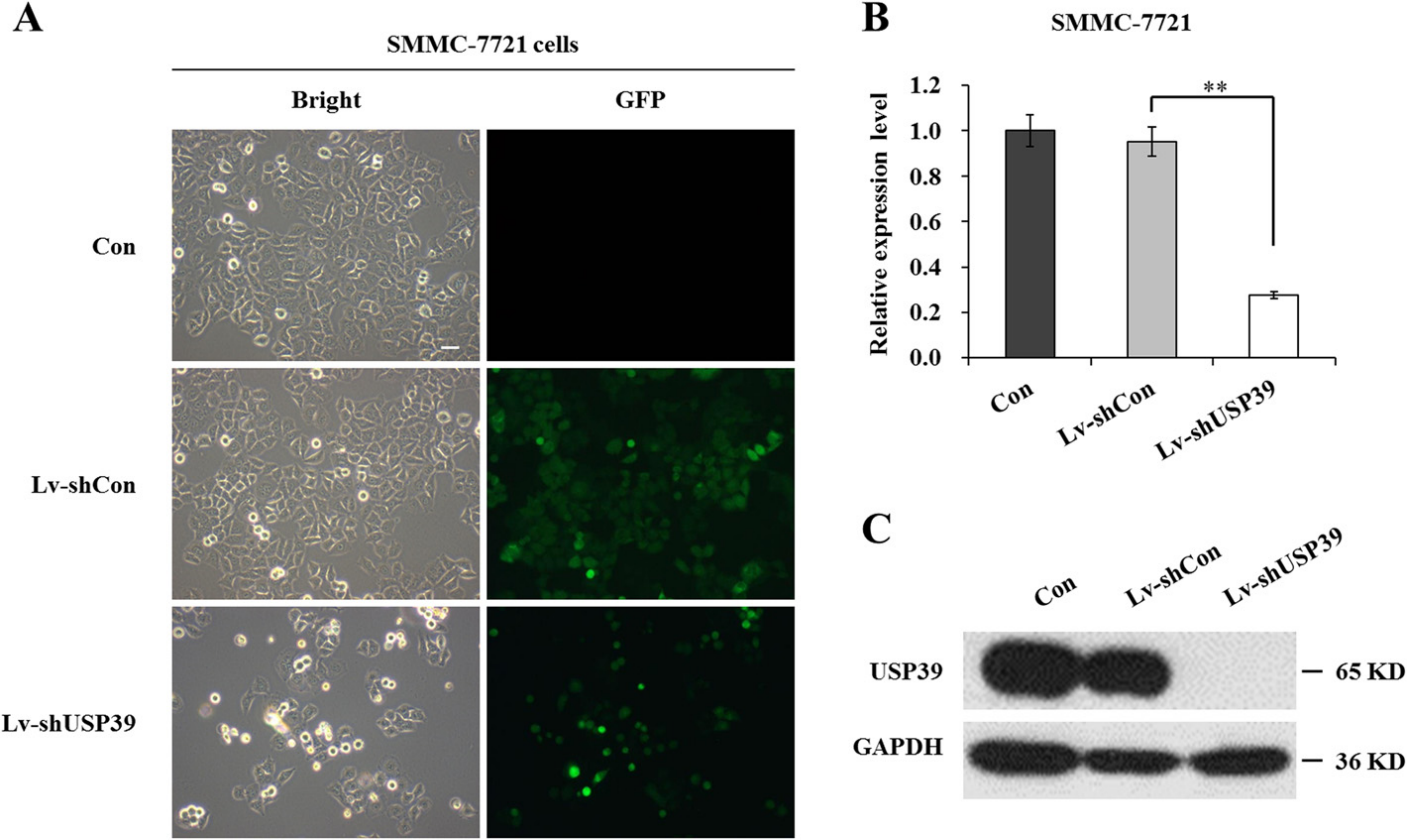
**Expression of USP39 is suppressed efficiently in SMMC-7721 cells after Lv-shUSP39 infection. (A)** Representative images of Con, Lv-shCon and Lv-shUSP39 infected SMMC-7721 cells under fluorescence microscope. Left, bright field; right, GFP. Scale bar, 10 μm. **(B)** qRT-PCR analyzed mRNA levels of USP39 in Con, Lv-shCon and Lv-shUSP39 infected SMMC-7721 cells. Actin was used as control gene. **, P < 0.01. **(C)** Western blotting analysis of protein levels of USP39 in in Con, Lv-shCon and Lv-shUSP39 infected SMMC-7721 cells. GAPDH was used as control protein.

To investigate the potential functions of USP39 in HCC, we knocked down USP39 in SMMC-7721 cells using lentivirus-mediated gene transfection. As shown in Figure 1A, most SMMC-7721 cells presented GFP-positive signals after infected by lentivirus recombined with shRNA targeting USP39 (Lv-shUSP39) or control scrambled shRNA (Lv-shCon), indicating that the recombinant lentivirus we got could infect SMMC-7721 cells with high efficiency. Further Real-time PCR and western-blot analysis suggested that the mRNA and protein levels of USP39 were both down-regulated significantly in Lv-shUSP39 infected SMMC-7721 cells (Figure 1B and C). The mRNA of USP39 was only 27% of that in control or Lv-shCon infected SMMC-7721 cells. No USP39 protein band was detected in Lv-shUSP39 infected cells. The above results indicated that recombinant lentivirus taking shUSP39 could effectively suppress the expression of endogenous USP39 in HCC cells.

### Down-regulation of USP39 inhibited cell proliferation and colony formation ability of SMMC-7721 cells

**Figure 2:**
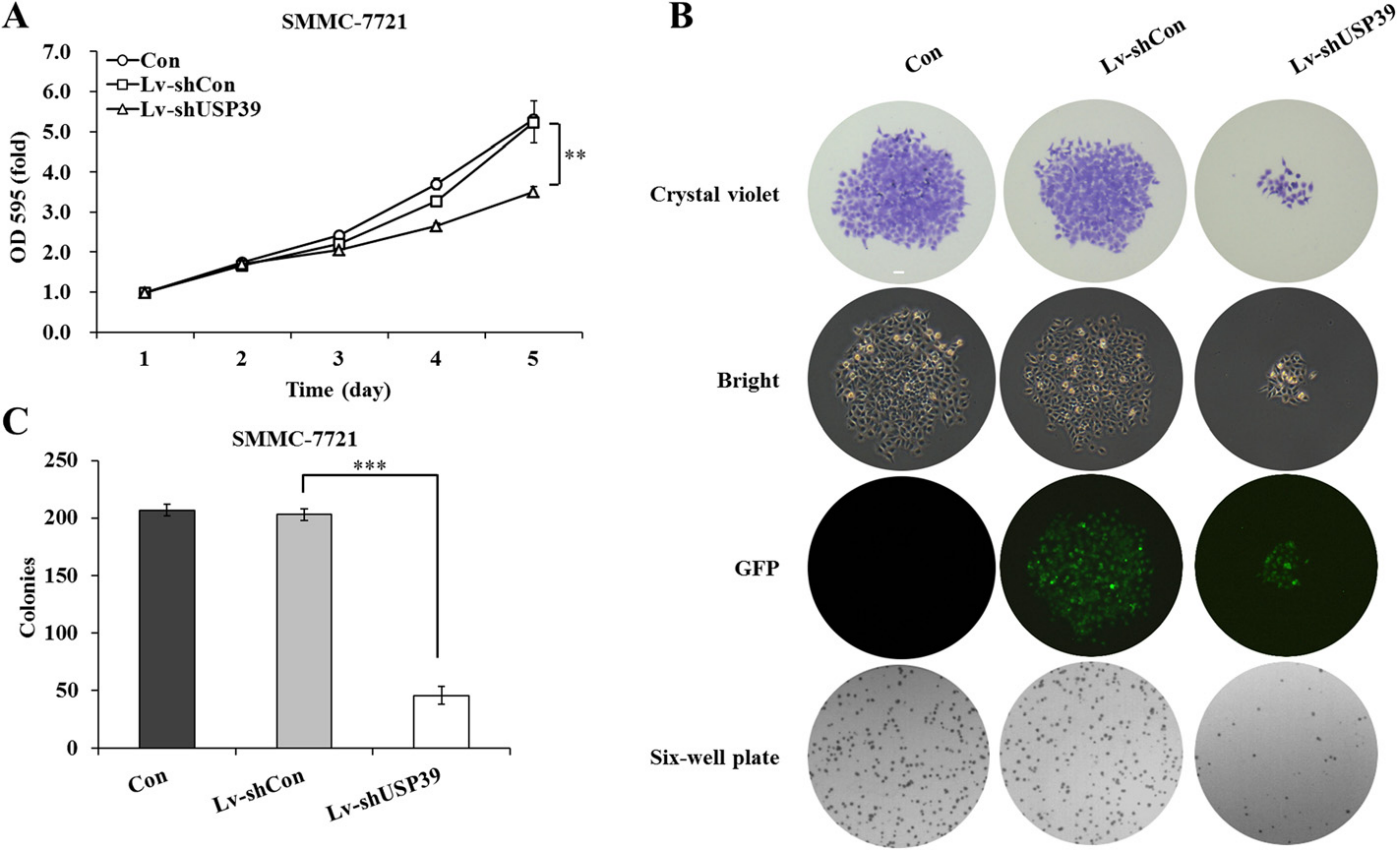
**Down-regulation of USP39 inhibits cell proliferation and colony formation ability of SMMC-7721 cells. (A)** The growth curves of Con, Lv-shCon and Lv-shUSP39 infected SMMC-7721 cells. **, P < 0.01. **(B)** Representative images of colony formation assays of Con, Lv-shCon and Lv-shUSP39 infected SMMC-7721 cells. From top to bottom: crystals violet staining, bright field and GFP of single colony, and crystals violet staining of six-well plate. Scale bar, 25 μm. **(C)** Colony numbers of Con, Lv-shCon and Lv-shUSP39 infected SMMC-7721 cells. ***, P < 0.001.

To study whether USP39 was related with SMMC-7721 cell proliferation, we performed 5-day MTT assay. Lv-shUSP39 infected SMMC-7721 showed slower growth rate compared with control and Lv-shCon infected cells (Figure 2A). On day 5, OD_595_ of Lv-shUSP39 infected cell was only 3.51 ± 0.12, while that of control and Lv-shCon infected cells were 5.31 ± 0.10 and 5.24 ± 0.53, respectively. We then analyzed the colony formation ability of SMMC-7721 cells after lentiviral infection using crystal violet staining. The cell number in a single colony was significantly reduced after Lv-shUSP39 infection (Figure 2B). Furthermore, we calculated the number of colons formed after lentivirus infection. The colony number of LvshUSP39 infected SMMC-7721 cells was only 46 ± 8, compared with that of 207 ± 5 in control cells and 203 ± 5 in Lv-shCon infected cells (Figure 2C). Furthermore, these results suggested that suppression of USP39 could inhibit cell proliferation and colony formation of HCC cells.

### Down-regulation of USP39 impaired cell cycle progression of SMMC-7721 cells

**Figure 3:**
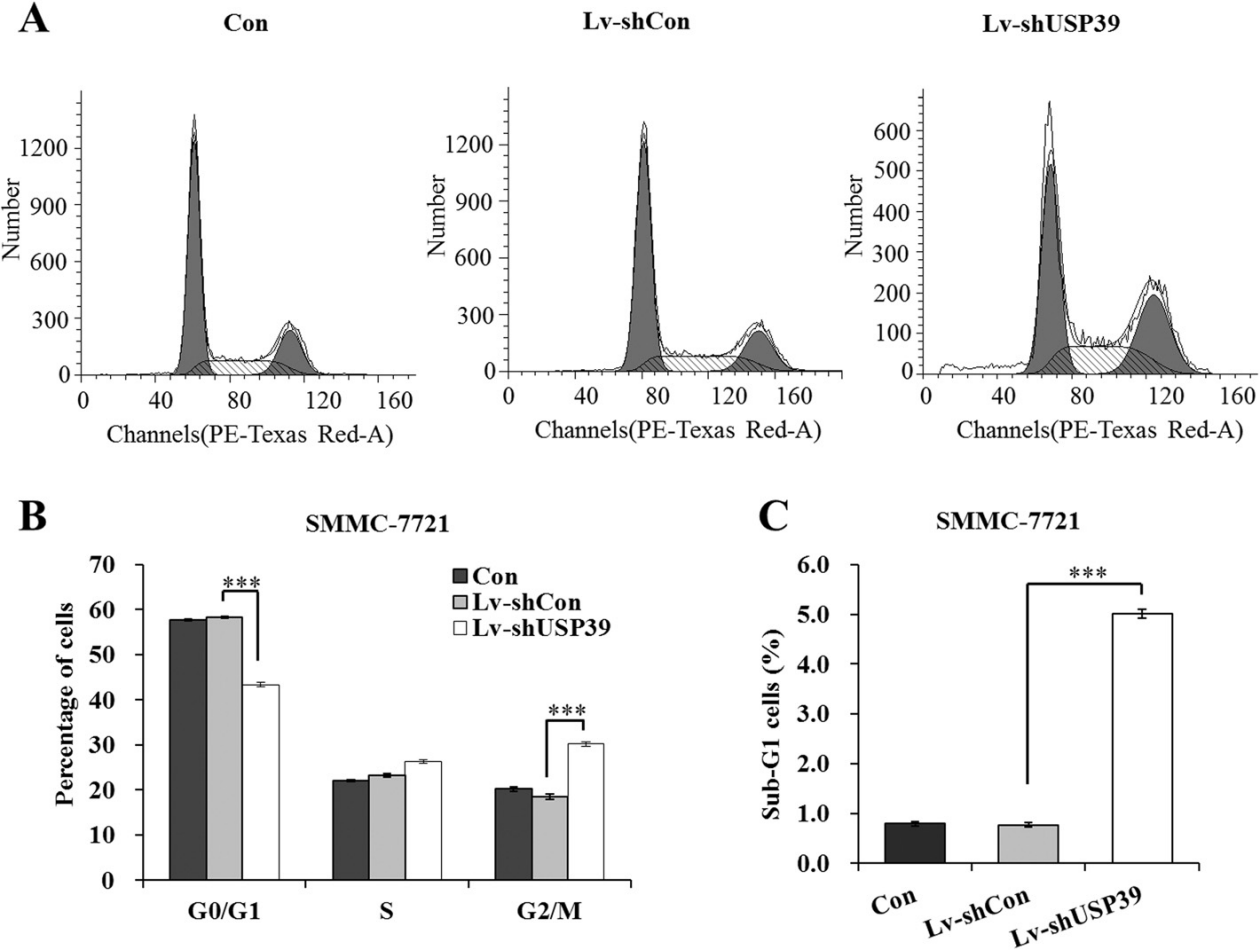
**Down-regulation of USP39 impairs cell cycle progression of SMMC-7721 cells. (A)** Representative graphs of flow cytometry analysis of SMMC-7721 cell cycle using PI staining. **(B)** Statistic analysis of percentages of SMMC-7721 cells at different cell cycle stages (G0/G1, S and G2/M). **(C)** Statistic analysis of percentages of sub-G1 SMMC-7721 cells. ***, P < 0.001.

To find out the underlying mechanisms of inhibition of cell proliferation and colony formation, we analyzed phases of cell cycle of SMMC-7721 cells after USP39 knockdown using flow cytometry with PI staining. As shown in Figure 3A and B, 43.44 ± 0.55% of cells were at G_0_/G_1_ phase in Lv-shUSP39 infected SMCC-7721 cells, which were significantly lower than those of control cells (57.78 ± 0.18%, p value) and Lv-shCon infected cells (58.28 ± 0.26%, p value). Meanwhile, there were more cells at G_2_/M phase after Lv-shUSP39 infection (30.20 ± 0.46%), compared with control cells (20.20 ± 0.43%) and Lv-shCon infected cells (18.52 ± 0.54%). These results indicated that cell cycle progression was impaired in USP39 knock-down HCC cells. In addition, 5.02 ± 0.09% cells were detected at sub-G_1_ phase in Lv-shUSP39 infected SMMC-7721 cells, much higher than those in control cells (0.79 ± 0.04%) and Lv-shCon infected cells (0.77 ± 0.05%) (Figure 3C), suggesting that there were more apoptotic cells after USP39 knock-down.

### Down-regulation of USP39 induced SMMC-7721 cells apoptosis

To further confirm the influence of USP39 on cell apoptosis, we used Annexin V-APC/7-AAD double staining on SMMC-7721 cells following lentivirus infection. Annexin V-APC vs 7-AAD plots from the gated cells showed the populations corresponding to viable (Annexin V-/AAD-), necrotic (Annexin V–/7-AAD+), early apoptotic (Annexin V+/7-AAD–), and late apoptotic (Annexin V+/7-AAD+) cells. The group of silencing USP39 increased the apoptotic cells (early apoptosis and late apoptosis) by 13-fold, as compared to the group of shCon (Additional file 1: Figure S1A and B). Therefore, These results demonstrated that USP39 gene interference induced a strong pro-apoptotic effect in human hepatocellular carcinoma SMMC-7721 cells. Thus, we may conclude that USP39 is involved in signaling pathways that intervene hepatocellular carcinoma cell apoptosis.

### Down-regulation of USP39 altered the expression of apoptotic proteins of SMMC-7721 cells

As shown in Figure 3C, knockdown of USP39 observably caused increased percentage of SMMC-7721 cells in the sub-G1 phase which represents apoptotic cells (p < 0.001). The expression change of apoptosis genes have been detected in SMMC-7721 cells after USP39 knockdown, including Bax, Caspase 9, Caspase 3 and PARP by western blot. It showed that knockdown of USP39 resulted in a significantly increase in the expressin of Bax, Caspase 9, Caspase 3 and PARP (Additional file 2: Figure S2). These results suggested that knockdown of USP39 in SMMC-7721 cells could induce cell apoptosis via altering the expression of Bax, Caspase 9, Caspase 3 and PARP.

## Discussion

Human hepatocellular carcinoma (HCC) is the most popular type of liver cancer, which is mainly caused by viral infection and is the third leading cause of cancer-related death [1-3]. It ranks as one of the top reasons of cancer related death all over the world [4,5]. The high rate of tumor recurrence and intrahepatic metastasis lead to poor prognosis of HCC patients [2]. RNAi, with the high specific and no apparent toxicity, is a promising gene therapeutic methods for the treatment of cancer in the furture [20]. Molecular targets are needed to be discovered for the treatment of hepatocellular carcinoma.

Cell growth and colony formation are two important indexes for tumorigenesis. Our studies indicate a defect of cell growth and colony formation of HCC cells after knock-down of USP39, which is similar to previous reports of USP39 in breast cancer cells [18]. Flow cytometry showed that down-regulation of USP39 resulted in arrest of SMMC-7721 cells at G_2_/M phase and more apoptosis. In consistent with our results, suppression of USP39 in MCF-7 cells leads to impaired cell cycle [18]. In addition, flow cytometry analysis using Annexin V-APC/7-AAD was applied to confirm the influence of USP39 on cell apoptosis. Downregulation of USP39 induced a remarkable pro-apoptotic effect in human hepatocellular carcinoma SMMC-7721 cells. Therefore, we could draw the conclusion that USP39 silencing led to the proliferation inhibition in human hepatocellular carcinoma presumably as a result of the induction of apoptosis. Furthermore, western blot showed that depletion of USP39 distinctly upregulated the expression of Bax, which pertains to the Bcl-2 family of pro-apoptotic proteins [21], and also augmented the protein expression levels of Caspase 9, Caspase 3 and cleaved PARP that are key apoptosis-associated proteins, especially the cleaved-PARP is considered to be a marker of apoptosis [22,23]. It further confirmed that knockdown of USP39 could suppress the growth of hepatocellular carcinoma cells through inducing cell apoptosis.

Sad1, the homologue of USP39 in yeast, could control multiple checkpoints of cell cycle [24]. Mutations of Sad1 affect both the spindle formation and function, as well as splicing [25,13]. Van Leuken *et al.* found that USP39 is a new factor required to maintain the spindle checkpoint and support successful cytokinesis through regulating of Aurora B mRNA splicing in mammalian cells [15]. Aurora B is a Ser/Thr kinase and supports the assembly of a functional bipolar spindle. Silencing the Aurora B using RNAi suppresses the activity of PI3K/Akt/NF-kB signaling pathway and inhibits proliferation, migration and invasion of various carcinoma cells [26-28]. Aurora B inhibitors have the antitumor activity and some of them have been tested in clinical trials in patients with solid tumors and acute myeloid leukemia [29-31]. In USP39 depleted U2OS cells, the mRNA level of Aurora B is dramatically reduced, which leads to defection in spindle checkpoint function and cytokinesis [15]. These evidences suggest USP39 might control cell proliferation through regulating Aurora B or other mRNA splicing.

One method to treat cancer is to induce apoptosis of cancer cells. Lv-shUSP39 infected SMMC-7721 cells showed increased sub-G_1_ phase cells, which are usually considered as an indicator of apoptotic fragmentation. USP39 knock-down MCF-7 cells present similar phenotype [18]. In addition, mutations of yeast Sad1is related with yeast cell viability and deletion of Sad1 is lethal [25]. Although lentivirus could efficiently deliver shRNA into mammalian cells and silencing USP39 in carcinoma cells, targeted gene delivery methods are need to be developed to specifically knockdown USP39 expression in tumor cells.

## Conclusions

In conclusion, our studies indicated that suppression of USP39 through recombinant lentivirus taking shUSP39 inhibited cell proliferation and colony formation of HCC cells along with cell cycle arrest at G_2_/M phase. These results suggest that USP39 may be a potential target gene for treatment of HCC.

## Methods

### Cell lines and culture conditions

HCC cell line SMMC-7721 and human embryonic kidney cell line 293 T (HEK293T) were obtained from Cell Bank of Chinese Academy of Sciences (Shanghai, China). Both of the cell lines were maintained in Dulbecco’s modified Eagle’s medium (DMEM, Hyclone, SH30243.01B+) plus 10% fetal bovine serum (FBS, Biowest, S1810) and 100 U/ml of penicillin-streptomycin. The cells were incubated at 37°C in a humidified atmosphere of 5% CO_2_. All experimental researches handling human cells were carried out in compliance with the Tenets of the Declaration of Helsinki.

### Lentivirus packaging and transduction

Short hairpin RNA (shRNA) targeting human USP39 gene (NM_001256725.1) was designed as following, 5′-CCTTCCAGACAACTATGAGATCTCGAGATCTCATAGTTGTCTGGAAGGTTTTT-3′. The control scrambled shRNA was 5′-GCGGAGGGTTTGAAAGAATATCTCGAGATATTCTTTCAAACCCTCCGCTTTTTT-3′. Both shRNAs were inserted into the vector pFH-L (Shanghai Hollybio, China) between restriction enzyme site *Nhe*I and *Pac*I. The shRNA taking plasmid together with two helper plasmids, pVSVG-I and pCMV∆R8.92 (Shanghai Hollybio, China), were transfected into the HEK293T cells using Lipofectamine 2000 (Life technologies, USA). Two days after transfection, cell culture media was collected and concentrated. The recombinant lentivirus was stored at -80°C.

SMMC-7721 cells were incubated in a six-well plate at an inoculation density of 5 × 10^4^ cells/well. The recombinant lentivirus containing media were added into the SMMC-7721 cells with a multiplicity of infection (MOI) of 30. Infection efficiency was determined through counting the numbers of GFP-positive cells under fluorescence microscope at 96 h after lentiviral infection.

### qRT-PCR

Primers 5′-GCCAGCAGAAGAAAAAGAGC-3′ (forward) and 5′- GCCATTGAACTTAGCCAGGA-3′ (reverse) were designed to detect USP39 expression. Actin was used as endogenous control and primers were 5′- GTGGACATCCGCAAAGAC -3′ (forward) and 5′- AAAGGGTGTAACGCAACTA -3′ (reverse). Six days after lentiviral infection, SMMC-7721 cells were harvested and total RNA was extracted using TRIzol (Life technologies, USA) according to the manufacturer’s instructions. Single strand cDNA was obtained using M-MLV reverse transcriptase kit (Promega, USA). USP39 mRNA level was then measured by qRT-PCR with SYBR master mixture (Applied Biosystems) on BioRad Connect Real-Time PCR platform. The 20 μl PCR reaction mixture was: 10 μL 2XSYBR premix ex taq, 0.8 μLprimers (2.5 μM), 5 μL cDNA and 4.2 μL ddH_2_O. The detailed PCR program was: initial denaturation at 95°C for 1 min; denaturation 95°C, 5 s; annealing extension of 60°C, 20 s (a total of 40 cycles). The absorbance values were read at the extension stage. Fold changes in expression have been calculated using the 2^-ΔΔCt^ method. Experiments were performed at least three times.

### Western blotting

Six days after lentiviral infection, SMMC-7721 cells were collected, washed with ice-cold PBS and lysed in 2× SDS sample buffer (100 mM Tris-Hcl (pH 6.8), 10 mM EDTA, 4% SDS, 10% Glycine). Equal amount of protein samples (30 μg) were run on the 10% SDS-PAGE gel at 50 V for 3 h. The proteins were then transferred to polyvinylidene fluoride (PVDF) membrane at 300 mA for 1.5 h. The membrane was blocked by 1% bovine serum albumin (BSA) in TBST at RT for 1 h and incubated with primary antibodies, mouse anti-USP39 (1:100, Sigma Aldrich, SAB1407042) , rabbit anti-PARP (1: 1000, Cell Signaling Technology, #9542), rabbit anti-Caspase 3 (1: 500, Cell Signaling Technology, #9661), rabbit anti- Caspase 9 (1:1000, Proteintech Group Inc., 10380-1-AP), rabbit anti-Bax (1: 500, Cell Signaling Technology, #2774) and rabbit anti-GAPDH (1:100,000, Proteintech Group Inc., 10494-1-AP) overnight at 4°C. After washed by TBST, membranes were incubated with horseradish peroxidase conjugated goat anti-mouse (1:5,000, Santa Cruz, SC-2005) and goat anti-rabbit (1:5,000, Santa Cruz, SC-2054) secondary antibodies for 2 h at room temperature. The membrane was washed by TBST and signals were detected by enhanced chemiluminescence kit (Pierce).

### Cell proliferation assay

Lentiviral infected SMMC-7721 cells were seeded in 96-well plates at an inoculation density of 3,000 cells/well. At different time points after incubation (1, 2, 3, 4 and 5 days), MTT (3-(4, 5-Dimethylthiazol-2-yl)-2, 5-diphenyltetrazolium bromide) solution was added to each well and incubated at 37°C for 4 h. Then 100 μL acidic isopropanol (10% SDS, 5% isopropanol and 0.01 mol/L HCl) was added into each well after the medium were carefully removed. Plates were then read at a wavelength of 595 nm. Experiments were performed at least three times.

### Colony formation assay

Lentiviral infected SMMC-7721 cells were seeded in 6-well plate at an initial density of 600 cells/well. Cells were cultured in 5% CO_2_ incubator for 8 days at 37°C. After wash by ice-cold PBS and fixation with 4% PFA, crystals purple staining was performed according to the instruction.

### Cell cycle assay

Lentiviral infected SMMC-7721 cells were seeded in 6 cm dishes at initial density of 80,000 cells/dish. After incubation at 37°C for 40 h, the cell confluence reached ~80%. SMMC-7721 cells were then collected, washed in cold PBS and fixed with pre-cold 75% ethanol. After digestion using RNase, the cells were stained with Propidium iodide (PI). Cell cycle distribution was then analyzed by using a flow cytometer (BD Biosciences). Experiments were performed at least three times.

### Cell apoptosis assay by flow cytometry

SMMC-7721 cells were subjected to three different treatments (Con, Lv-shCon, and Lv-shUSP39). After 96 h of incubation, cells were washed by PBS and re-seeded in 6 cm dishes at a density of 8 × 104 cells/dish before detection. After 48 h, the cells were collected, washed with PBS for three times and subjected to Annexin V-APC/7-AAD double staining according to the instruction of Apoptosis Assays Kit (KeyGEN Biotech, Nanjing, China). Flow cytometry analysis was performed on FACS caliber II sorter and Cell Quest FACS system (BD Biosciences, USA).

### Statistical analysis

Comparisons between groups were done using paired Student’s t test, and differences were considered statistically significant at P < 0.05. Data were presented as mean ± standard deviation (SD) of three independent experiments.

### Ethical standards

This study was also reviewed and approved by the Institutional Review Board of Eastern Hepatobiliary Hospital.

## Additional files

Additional file 1: Figure S1. Down-regulation of USP39 induced SMMC-7721 cells apoptosis. A. Cytogram of Annexin V-APC binding vs. 7-AAD uptake in SMMC-7721 cells (Con, Lv-shCon and Lv-shGPR137). B. Percentage of apoptotic cells in SMMC-7721 cells by FACS. Annexin V-/7-AAD-: viable cells, Annexin V-/7-AAD+: necrotic cells, Annexin V+/7-AAD-: early apoptotic cells; Annexin V+/7-AAD+: late apoptotic cells. Additional file 2: Figure S2. Down-regulation of USP39 altered the expression of apoptotic proteins of SMMC-7721 cells. Western blotting analysis of apoptosis protein expression levels of Bax, Caspase 9, Caspase 3 and PARP in in Con, Lv-shCon and Lv-shUSP39 infected SMMC-7721 cells. GAPDH was used as control protein.
