# Expression of Concern to: Lentivirus mediated silencing of Ubiquitin Specific Peptidase 39 inhibits cell proliferation of human hepatocellular carcinoma cells in vitro

**DOI:** 10.1186/s40659-018-0170-y

**Published:** 2018-06-22

**Authors:** Zeya Pan, Hao Pan, Jin Zhang, Yun Yang, Hui Liu, Yuan Yang, Gang Huang, Junsheng Ni, Jian Huang, Weiping Zhou

**Affiliations:** 1 The Third Department of Hepatic Surgery, Eastern Hepatobiliary Surgery Hospital, Second Military Medical University, 200438 Shanghai, China; 2 Department of Infectious Disease Control and Prevention, Shanghai Municipal Center for Disease Control and Prevention, 1380 West Zhongshan Road, 200336 Shanghai, China

## Expression of Concern: Biol Res (2015) 48:18 10.1186/s40659-015-0006-y

Concerns have been raised about this article [1] relating to the appropriateness of the use of the shRNA (5′-GCGGAGGGTTTGAAAGAATATCTCGAGATATTCTTTCAAACCCTCCGCTTTTTT-3′) as a non-targeting control and similarities in text and formatting with other published articles. This is currently under investigation and appropriate editorial action will be taken once the investigation is concluded. The authors agree.
