# Supplementary figures and images for: Lentivirus mediated silencing of Ubiquitin Specific Peptidase 39 inhibits cell proliferation of human hepatocellular carcinoma cells in vitro
Source: Biol Res. 2015 Mar 19;48(1):18. doi: 10.1186/s40659-015-0006-y (PMC4389921; doi:10.1186/s40659-015-0006-y)

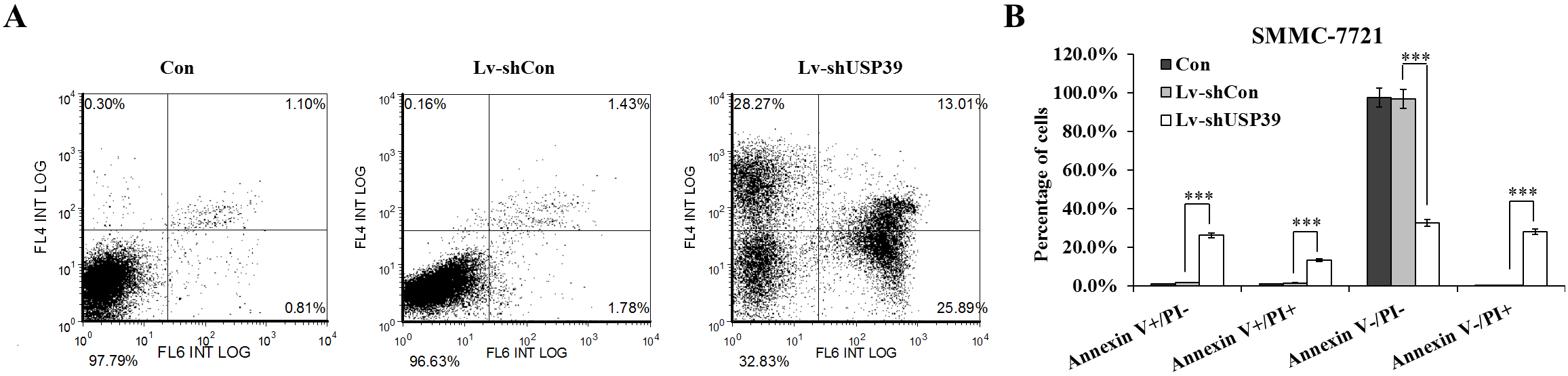

Supplement: Additional file 1: Figure S1. — Down-regulation of USP39 induced SMMC-7721 cells apoptosis. A. Cytogram of Annexin V-APC binding vs. 7-AAD uptake in SMMC-7721 cells (Con, Lv-shCon and Lv-shGPR137). B. Percentage of apoptotic cells in SMMC-7721 cells by FACS. Annexin V-/7-AAD-: viable cells, Annexin V-/7-AAD+: necrotic cells, Annexin V+/7-AAD-: early apoptotic cells; Annexin V+/7-AAD+: late apoptotic cells. [file 40659_2015_6_MOESM1_ESM.tiff]

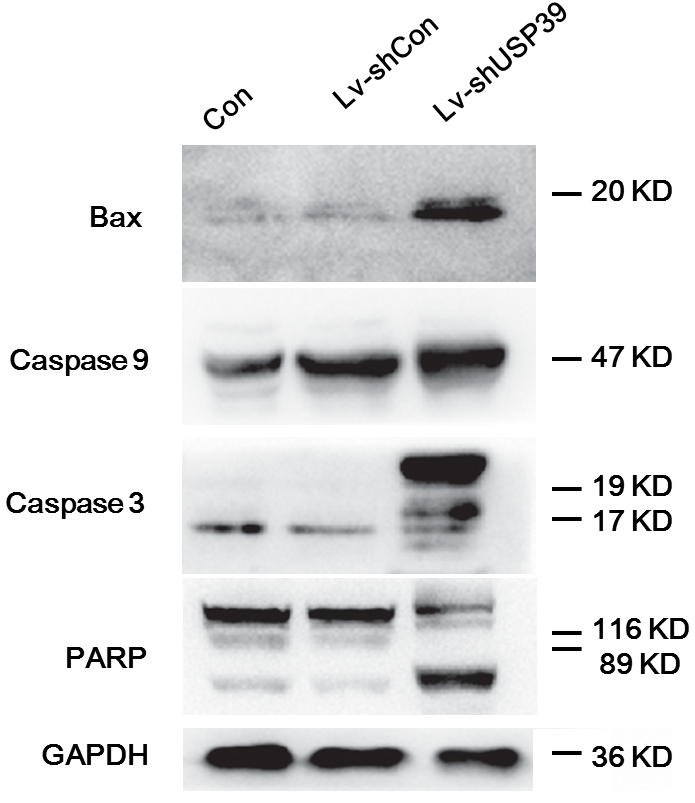

Supplement: Additional file 2: Figure S2. — Down-regulation of USP39 altered the expression of apoptotic proteins of SMMC-7721 cells. Western blotting analysis of apoptosis protein expression levels of Bax, Caspase 9, Caspase 3 and PARP in in Con, Lv-shCon and Lv-shUSP39 infected SMMC-7721 cells. GAPDH was used as control protein. [file 40659_2015_6_MOESM2_ESM.tiff]
